# Triazole Susceptibilities in Thermotolerant Fungal Isolates from Outdoor Air in the Seoul Capital Area in South Korea

**DOI:** 10.1371/journal.pone.0138725

**Published:** 2015-09-25

**Authors:** Seungeun Lee, Siyu Xu, Chemmeri Padasseri Bivila, Hyeyoung Lee, Myung Soo Park, Young Woon Lim, Naomichi Yamamoto

**Affiliations:** 1 Department of Environmental Health Sciences, Graduate School, of Public Health, Seoul National University, Seoul 151–742, South Korea; 2 School of Biological Sciences, College of Natural Sciences, Seoul National University, Seoul 151–742, South Korea; Woosuk University, REPUBLIC OF KOREA

## Abstract

Emerging fungi resistant to triazoles are a concern because of the increased use of medical triazoles and exposure to agricultural triazoles. However, little is known about the levels of triazole susceptibility in outdoor airborne fungi making it difficult to assess the risks of inhalation exposure to airborne, antifungal-resistant fungi. This study examined triazole susceptibilities of the airborne thermotolerant fungi isolated from the ambient air of the Seoul Capital Area of South Korea. We used impactor air sampling with triazole-containing nutrient agar plates as the collection substrates to screen for airborne fungal isolates based on their triazole susceptibilities. This study estimated that 0.17% of all the culturable fungi belong to the pathogenic thermotolerant taxa, among which each isolate of *Aspergillus niger* and *Aspergillus tubingensis* showed a minimum inhibitory concentration (MIC) of 2 μg/mL or greater for itraconazole. Their concentration in air was 0.4 CFU/m^3^. Seven human pathogenic *Paecilomyces variotii* isolates had MICs of 32 μg/mL or greater and lower than 2 μg/mL for the agricultural fungicide tebuconazole and the medical triazole itraconazole, respectively. Though the concentration was low, our results confirm the presence of airborne fungi with high MICs for itraconazole in ambient air. Inhalation is an important exposure route because people inhale more than 10 m^3^ of air each day. Vigilance is preferred over monitoring for the emergence of triazole-resistant fungal pathogens in ambient outdoor air.

## Introduction

Fungi are ubiquitous in environments such as soil [1–3], house dust [4, 5], human skin [6], and air [7–10]. While most fungi are harmless to humans, some are infectious pathogens. For instance, *Aspergillus* spp. can cause invasive fungal infections (IFI) with a fatality ratio of approximately 60% reported for immunocompromised individuals [11]. *Aspergillus fumigatus*, a primary and opportunistic fungal pathogen [12], produces small unicellular spores with a reported diameter of 2 to 3.5 μm [13]. These spores are easily released into the air and inhaled and partly respired by humans [14]. Inhaled *Aspergillus* spores in the human respiratory system can cause invasive pulmonary aspergillosis (IPA) in immunocompromised individuals [15]. Because of the high fatality rate of IPA [16], proper treatment is crucial.

Triazoles are a group of antifungal chemicals which are often administered to IFI patients [16, 17]. Triazoles, which are organic compounds with a five-member ring consisting of two carbon and three nitrogen atoms, are used as medical drugs as well as agricultural fungicides. Medical triazoles include itraconazole, voriconazole, and posaconazole [16, 17], whereas agricultural triazoles include tebuconazole, difenoconazole, and propiconazole [18, 19]. Triazoles are considered to be the most successful class of antifungal drugs in clinics [20]; however, there is concern over the emergence of resistant strains. One study showed that there is an increasing frequency of itraconazole-resistant *Aspergillus*, in which the resistance developed in the bodies of the patients because of the treatment failure [21]. Moreover, triazole-resistant *A*. *fumigatus* has been detected in various environments including indoor air and on surfaces in hospitals [22–24].

Agricultural triazoles are also a concern because they create selective pressures for environmental fungi resistant to triazoles [25]. One study showed a cross-resistance between medical and agricultural triazoles in *A*. *fumigatus* [26], indicating the possibility of strains resistant to medical triazoles having increased environmental fitness over wild-type strains under the selective pressure of agricultural triazoles. Accumulating evidence has shown that there is an emerging *A*. *fumigatus* resistant to medical triazoles found in environments such as compost [27], soil [22, 23, 27, 28], and outdoor air [22, 24]. In South Korea, residual triazole fungicides (e.g., tebuconazole) have been detected in dried vegetables [29], which is a good indicator of the nationwide use of agricultural triazoles. Thus, vigilance is needed in terms of the emergence of fungal strains in the environment with resistance to agricultural and medical triazoles.

The aim of this study was to investigate the level of triazole susceptibility in fungal isolates from ambient outdoor air in the Seoul Capital Area of South Korea. This study particularly focused on thermotolerant fungi because they are more likely to be infectious than that of other mesophilic fungi [30]. We did seasonal air sampling with an impactor sampler and triazole-containing nutrient agar plates, followed by incubation at 35°C. The testing of susceptibility to triazoles was done with the standard broth dilution method. We tested the triazoles itraconazole and tebuconazole because cross-resistance to both medical and agricultural triazoles is of great concern [26]. At present, little is known about the levels of triazole susceptibility in outdoor airborne thermotolerant fungi [22–24]. This study provides insight into how triazole resistance is prevalent in thermotolerant fungi in ambient outdoor air.

## Materials and Methods

### Air sampling

Air sampling was conducted from November 2013 to October 2014 at three different locations in the Seoul Capital Area of South Korea. One location was at the campus of Seoul National University at Gwanak-gu, Seoul (37°27'55.0"N; 126°57'17.7"E) and the other two locations at a test farm belonging to the university in Suwon, Gyeonggi-do, South Korea which included an apple field (37°15'36.0"N; 126°58'57.6"E) and a rice field (37°16'12.5"N; 126°59'20.6"E). The test farm is located 21.9 km south of the university campus. On the test farm, the apple and rice fields are separated by a distance of 1.2 km. Typically, air sampling was conducted bimonthly at each location alternating between either the university campus or the test farm. A total of 21 sets of air sampling were collected: nine sets at the university campus and twelve sets at the test farm (six sets each for the apple and rice fields). Prior to conducting the field studies, all the necessary permits were obtained from the managers at each site.

A single-stage multi-jet impactor sampler (Quick Take 30, SKC Inc., Eighty Four, PA, USA) was used to conduct the air sampling. Sabouraud dextrose agar plates containing chloramphenicol (SDAC) and Roswell Park Memorial Institute (RPMI) agar plates were used as the impactor substrates. SDAC plates containing 0.005% (w/w) chloramphenicol (Hanil Komed Co., Ltd., Seongnam, South Korea) were used to quantify the total culturable fungi while RPMI plates were used to quantify the thermotolerant fungi. One liter of RPMI agar plate medium consisted of 10 g of RPMI 1640 medium powder (Gibco Life Technologies, Carlsbad, CA, USA), 35 g of morpholinepropanesulfonic acid buffer (MOPS; Dojindo, Tokyo, Japan), 15 g of agar (AMRESCO, Solon, OH, USA), and 0.005% (w/w) chloramphenicol (Hanil Komed Co., Ltd.). To quantify the susceptibility of thermotolerant fungi to triazoles, 0, 0.0625, 1, 4, or 16 μg/mL of itraconazole (Santa Cruz Biotechnology, Santa Cruz, CA, USA) or tebuconazole (Santa Cruz Biotechnology, Inc.) were added to the RPMI agar [31]. A total of twelve agar plates (1 SDAC and 11 RPMI agar plates) with or without triazoles were used for each set of air sampling.

Airborne fungi were collected on each agar plate for 2 or 5 min with an air flow rate of 28.3 L/min. For each set of air sampling, twelve consecutive air samplings were performed using agar plates containing each concentration of the triazoles. Typically, a duration of 2 min was chosen for air sampling with the SDAC plates because a large number of total culturable fungi was expected in the spring, summer, and fall. A shorter sampling duration was used to avoid overloading the plates with fungal particles. Meanwhile, a duration of 5 min was used for the air sampling with the RPMI plates because a smaller number of thermotolerant fungi were expected, thus requiring a longer sampling duration. The exposed plates were transported to the laboratory within 8 h from the start of the air sampling and incubated so that the fungal colony-forming units (CFU) could be counted. The incubation temperatures were 25°C and 35°C for the SDAC and RPMI plates, respectively. Positive-hole corrections were made for all reported airborne fungal concentrations to correct for the possibility that multiple viable particles passed through the same sampling hole of the multi-jet impactor and were deposited together at the same point on the collection substrate [32].

### DNA sequencing

Fungal colonies identified on the agar plates containing 4 and 16 μg/mL of ITZ or TBZ were isolated, and pure cultures were obtained by isolating individual colonies onto new plates. These isolates from the pure cultures were used for DNA sequence-based analyses and standard susceptibility testing. DNA was extracted from pure culture colonies from an area approximately 0.25 cm^2^ in size using the OmniPrep for Fungi kit (Geno Technology Inc., St. Louis, MO, USA) following the manufacturer’s instructions. Briefly, fungal tissues were grinded with the grinding resin of the kit and zirconia beads. The resultant cellular debris and proteins were removed by precipitation using the included reagents of the kit. DNA pellets were obtained with isopropanol precipitation and rehydrated in TE for storage at -20°C until the subsequent analyses.

These isolates were identified based on the sequences of the nuc-rDNA internal transcribed spacer (ITS; ITS1-5.8S-ITS2) and β-tubulin (*benA*) gene. The ITS sequences were amplified with the forward primer ITS1F (5’-CTTGGTCATTTAGAGGAAGTAA-3’) and the reverse primer ITS4 (5’-TCCTCCGCTTATTGATATGC-3’) [33]. The *benA* sequences were amplified with Bt2a (5’-GGTAACCAAATCGGTGCTGCTTTC-3’) and Bt2b (5’-ACCCTCAGTGTAGTGACCCTTGGC-3’) [34]. Each PCR mixture (total volume of 30 μL) had 1 μL of template DNA, 1× PCR Master Mix (Takara Bio Inc., Shiga, Japan), and 0.3 μM of each primer. The amplification process was done under the following thermal conditions: an initial denaturation step of 95°C for 5 min and then 35 cycles of denaturation at 95°C for 30 s, annealing at 55°C for 30 s, and extension at 72°C for 1 min followed by a final extension at 72°C for 10 min. PCR amplicons were purified with the HiGene PCR purification kit (SolGent Co., Ltd., Daejeon, Korea). PCR amplicons were sequenced with the ABI3730xL Genetic Analyzer (Life Technologies, Carlsbad, CA, USA). The lengths of the sequenced amplicons were from 603 to 705 bp for the ITS sequences and from 507 to 534 bp for the *benA* sequences.

To identify the isolates at the species level, the ITS and *benA* sequences of each isolate were compared with the sequences of type strains downloaded from GenBank. A species was assigned to an isolate if a single type-strain sequence matched the sequence of the isolate with more than 99% sequence-homology using BLASTn; otherwise, isolates were defined as ‘unidentified’ if multiple type-strain sequences matched with the same e-values as the top hit.

For the isolates identified as *Aspergillus fumigatus*, the full coding sequence of the *cyp51A* gene and the promoter region were further analyzed for their mutations [24, 25, 35–37]. The coding and promoter regions of the *cyp51A* gene were amplified with two primer sets: P450-A1 (5’-ATGGTGCCGATGCTATGG-3’) and P450-A2 (5’-CTGTCTCACTTGGATGTG-3’) [35], and P-A7 (5’-TCATATGTTGCTCAGCGG-3’) and P-A5 (5’-TCTCTGCTGCACGCAAAGAAGAAC-3’) [38]. The same PCR reagents described above were used, and PCR was performed with a thermal cycle of 94°C for 5 min followed by 30 cycles of 94°C for 30 s, 58°C for 45 s, and 72°C for 2 min and a final 10 min extension at 72°C. The purification and sequencing of the amplicons were done as described above. Full-length *cyp51A* gene coding sequences were produced with an internal primer (5’-CGC ACATGATGATAACCC-3’) and assembled with the promoter region sequence using the SeqMan Pro Lasergene suite (DNASTAR Inc., Madison, WI, USA). To identify mutations in the *cyp51A* genes, the assembled sequences were compared with a reference sequence from wild-type *A*. *fumigatus* AF338659.1 [39]. All *A*. *fumigatus* isolates analyzed were wild type with no mutations in the *cyp51A* gene and no variants in the 34-bp tandem repeat in the promoter region.

The sequencing data of the *benA*, ITS, and *cyp51A* gene with the promoter sequences were deposited in GenBank with accession numbers of KP724980-724984, KP724985-725001, and KP725002-725004, respectively.

### Susceptibility testing

Susceptibility testing was done for all fungi identified on the plates containing 4 and 16 μg/mL of ITZ or TBZ in accordance with the Clinical and Laboratory Standards Institute (CLSI) M38-A2 broth dilution protocol [40]. Briefly, the tested triazole concentrations ranged from 0.03 to 32 μg/mL. The minimum inhibitory concentrations (MICs) were determined for each inoculum of the isolates with a spore suspension adjusted to range from 0.4×10^4^ to 5×10^4^ CFU/mL. Each inoculum was incubated at 35°C for 48 h. For each isolate, duplicate experiments were done twice, that is, a total of four experiments. The median of the four MICs obtained for each isolate was used to report the final MIC. Quality control was done each day using the two reference strains recommended by the CLSI: *Candida parapsilosis* ATCC 22019 and *Candida krusei* ATCC 6258. Additionally, four *Aspergillus* strains, *Aspergillus flavus* ATCC 204304 and *Aspergillus fumigatus* ATCC MYA 3626, ATCC MYA 4609 and ATCC MYA 3627, were included to ensure the accuracy of the susceptibility tests.

## Results

### Concentrations of the total airborne and thermotolerant fungi

**Fig 1:**
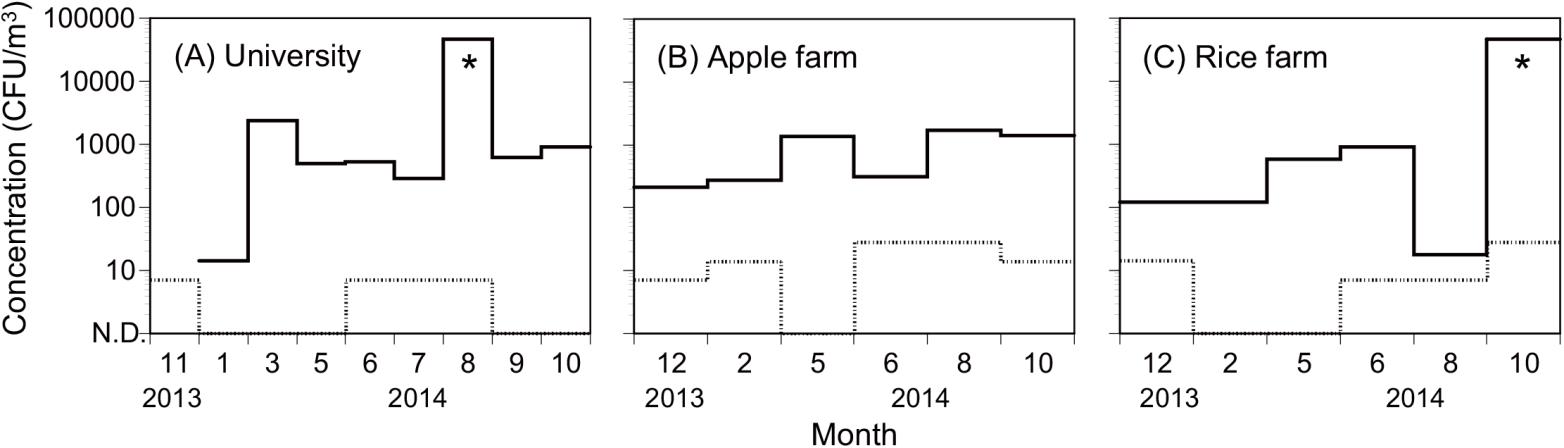
Seasonal concentrations of airborne total culturable (solid lines) and thermotolerant (dashed lines) fungi. (A) university campus, (B) apple field, and (C) rice field in the capital area of Seoul, South Korea. Asterisks indicate data above the upper limit of quantification (>46,000 CFU/m^3^).

Fig 1 shows the seasonal concentrations of the total airborne culturable and thermotolerant fungi at three locations in the Seoul Capital Area of South Korea. The airborne concentrations of total culturable fungi grown on the SDAC plates incubated at 25°C were higher than the concentrations of the thermotolerant fungi grown on the non-triazole-containing RPMI plates incubated at 35°C. Substantial seasonal variations were observed in the airborne concentrations of total culturable fungi, ranging from 14 to >46,000 CFU/m^3^. The concentrations of airborne thermotolerant fungi ranged from 0 to 28 CFU/m^3^.

### Number of fungal colonies observed on the plates for each concentration of the triazoles

**Fig 2:**
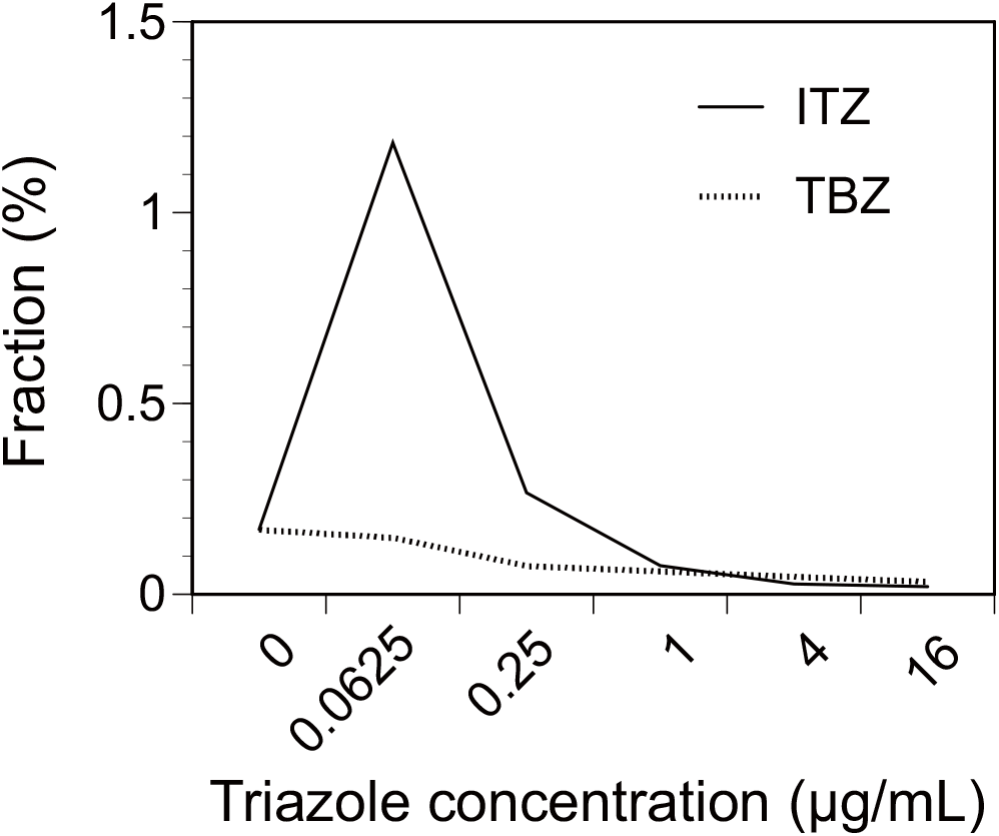
Fractions of the number of fungal colonies observed on RPMI plates containing each triazole concentration. The fractions of the number of fungal colonies observed on the RPMI plates for each triazole concentration to the number of fungal colonies observed on the SDAC plates are shown. The cumulative data for all sampling months and locations are used. Abbreviations: ITZ, itraconazole; TBZ, tebuconazole.

A total of 1,611 CFUs were observed on the SDAC plates incubated at 25°C (Table 1). The CFUs on the SDAC plates were set as the concentration of total culturable fungi [41, 42], while the CFUs on the non-triazole-containing RPMI plates incubated at 35°C were set as the concentrations of total thermotolerant fungi. A total of 25 thermotolerant fungal colonies were detected on the non-triazole-containing RPMI plates (Table 1), representing 0.17% of the total culturable fungi after positive-hole correction and adjustment for differences in the sampling durations across each air sampling (2 or 5 min) (Fig 2). Notably, the fractions were greater for the plates containing 0.0625 and 0.25 μg/mL of ITZ, i.e., 1.18% and 0.27%, respectively (Fig 2). However, the fractions were smaller for the higher ITZ concentrations, i.e., 0.07%, 0.03%, and 0.02% for 1, 4, and 16 μg/mL, respectively. The fractions for the TBZ-containing plates were all smaller than those of the non-triazole-containing plates, i.e., 0.15%, 0.07%, 0.06%, 0.05%, and 0.03% for 0.0625, 0.25, 1, 4, and 16 μg/mL, respectively (Fig 2). Nineteen colonies in total were isolated from the plates containing 4 or 16 μg/mL of ITZ or TBZ (Table 1), for which subsequent susceptibility testing and sequencing-based analyses were performed.

**Table 1:** Fungal colony forming units (CFUs) observed on each nutrient agar plate^a^.

Sampling site	Day (mm/dd/yyyy)	SDAC ^b^	RPMI without triazole ^c^	RPMI with ITZ (μg/mL) ^c^					RPMI with TBZ (μg/mL) ^c^				
				0.0625	0.25	1	4	16	0.0625	0.25	1	4	16
University	11/11/2013	n.a.	1	0	1	0	0	0	1	0	0	0	0
	01/10/2014	2	0	2	1	0	0	0	0	0	0	0	0
	03/14/2014	229	0	0	0	2	0	1	0	1	1	0	0
	05/23/2014	27*	0	54	1	0	0	0	0	0	0	0	0
	06/13/2014	29*	1	1	3	0	0	0	0	1	0	0	5
	07/03/2014	16*	1	2	2	2	0	0	1	0	0	0	0
	08/11/2014	400*	1	2	4	0	0	0	1	0	1	0	0
	09/12/2014	34*	0	0	0	0	0	0	1	0	0	1	0
	10/06/2014	48*	0	0	0	0	0	0	0	0	0	1	0
Apple farm	12/13/2013	29	1	0	0	1	0	0	1	3	0	0	0
	02/21/2014	37	2	0	2	0	0	0	2	1	0	0	0
	05/16/2014	71*	0*	0*	0*	0*	0*	0*	0*	0*	0*	0*	0*
	06/30/2014	17*	4	1	0	0	0	0	0	0	0	0	0
	08/05/2014	85*	4	90	4	4	3	2	6	1	2	2	0
	10/08/2014	72*	2	0	1	0	0	0	3	0	1	1	0
Rice farm	12/13/2013	17	2	5	1	0	0	0	4	0	1	1	0
	02/21/2014	17	0	0	1	0	0	0	0	0	1	0	0
	05/16/2014	32*	0*	0*	0*	0*	0*	0*	0*	1*	0*	0*	0*
	06/30/2014	48*	1	0	1	0	0	0	0	2	1	0	0
	08/05/2014	1*	1	0	7	1	0	0	0	1	0	1	0
	10/08/2014	400*	4	1	10	1	1	0	2	0	1	0	0
Total		1611	25	158	39	11	4	3	22	11	9	7	5

^a^ The positive-hole corrections are not made for these raw CFU values.

^b^ Incubated at 25°C.

^c^ Incubated at 35°C.

Symbol: *, Data of 2-min air samplings; No asterisk, data of 5-min air samplings.

### Fungal species observed on plates containing 4 or 16 μg/mL of the triazoles

Based on the ITS and *benA* sequences, seven fungal species were identified from a total of 19 isolates from the plates containing 4 or 16 μg/mL of ITZ or TBZ (Table 2). Three fungal isolates were not identifiable, for which susceptibility testing was not done. The species identified were *Aspergillus fumigatus*, *Aspergillus niger*, *Aspergillus tubingensis*, *Choanephora infundibulifera*, *Coprinellus curtus*, *Coprinopsis cinerea*, and *Paecilomyces variotii*. Susceptibility testing could not be done for the three non-Ascomycota fungi, *Coprinellus curtus*, *Coprinopsis cinerea*, and *Choanephora infundibulifera*, because of difficulty inducing sporulation. Consequently, twelve isolates from four species, *A*. *fumigatus*, *A*. *niger*, *A*. *tubingensis*, and *P*. *variotii*, were analyzed with susceptibility testing.

**Table 2:** Minimum inhibitory concentrations (MICs) of ITZ and TBZ in the airborne thermotolerant fungal isolates determined by the CLSI M38-A2 broth dilution method.

Type	Species	Strain ID	GenBank accession number (ITS/*benA*/*cyp51A*)	Location	Day (mm/dd/yyyy)	Type of agar plate (concentration, μg/mL) ^a^	ITZ MIC (μg/mL) ^b^	TBZ MIC (μg/mL) ^b^
Sampled strains								
	*Aspergillus niger*	13L06I1	KP724986/KP24980	University	03/14/2014	ITZ (16)	2.0	4.0
	*Paecilomyces variotii*	17L16T1	KP724987	University	06/13/2014	TBZ (16)	0.75	≥32
	*Paecilomyces variotii*	17L16T2	KP724988	University	06/13/2014	TBZ (16)	0.75	≥32
	*Paecilomyces variotii*	17L16T3	KP724989	University	06/13/2014	TBZ (16)	0.75	≥32
	*Paecilomyces variotii*	17L16T4	KP724990	University	06/13/2014	TBZ (16)	1.0	≥32
	*Paecilomyces variotii*	17L16T5	KP724991	University	06/13/2014	TBZ (16)	1.0	≥32
	*Aspergillus fumigatus*	24L15T1	KP724998/KP24983/KP725004	University	09/12/2014	TBZ (4)	1.5	2.0
	*Paecilomyces variotii*	25L15T1	KP724999	University	10/06/2014	TBZ (4)	0.75	≥32
	*Aspergillus fumigatus*	21L05I1	KP724992/KP24981/KP725002	Apple farm	08/05/2014	ITZ (4)	1.5	2.0
	*Aspergillus fumigatus*	21L05I2	KP724993/KP24982/KP725003	Apple farm	08/05/2014	ITZ (4)	1.0	2.0
	*Coprinellus curtus*	21L05I3	KP724994	Apple farm	08/05/2014	ITZ (4)	n.d.	n.d.
	*Coprinopsis cinerea*	21L06I1	KP724995	Apple farm	08/05/2014	ITZ (16)	n.d.	n.d.
	*Coprinopsis cinerea*	21L06I2	KP724996	Apple farm	08/05/2014	ITZ (16)	n.d.	n.d.
	Unidentified	21L15T1		Apple farm	08/05/2014	TBZ (4)	n.d.	n.d.
	*Choanephora infundibulifera*	21L15T2	KP724997	Apple farm	08/05/2014	TBZ (4)	n.d.	n.d.
	*Paecilomyces variotii*	26L15T1	KP725000	Apple farm	10/08/2014	TBZ (4)	1.0	≥32
	Unidentified	05L15T1	KP724985	Rice farm	12/13/2013	TBZ (4)	n.d.	n.d.
	Unidentified	22L15T1		Rice farm	08/05/2014	TBZ (4)	n.d.	n.d.
	*Aspergillus tubingensis*	27L05I1	KP725001/KP24984	Rice farm	10/08/2014	ITZ (4)	4.0	8.0
Quality control strains								
	*Candida parapsilosis*	ATCC 22019	-	-	-	-	0.25 [0.12–0.5] ^c^	1.0
	*Candida krusei*	ATCC 6258	-	-	-	-	0.5 [0.25–1.0] ^c^	4.0
Reference strains								
	*Aspergillus flavus*	ATCC 204304	-	-	-	-	0.25 [0.25–0.5] ^c^	1.0
	*Aspergillus fumigatus*	ATCC MYA 3626	-	-	-	-	1.0 [0.25–2.0] ^c^	2.0
	*Aspergillus fumigatus*	ATCC MYA 4609	-	-	-	-	1.0	4.0
	*Aspergillus fumigatus*	ATCC MYA 3627	-	-	-	-	≥32 [>16] ^c^	4.0

^a^ Types of agar plates on which the strains were isolated.

^b^ Median values of at least four replicated experiments were used.

^c^ The values in brackets are the MIC ranges reported by the CLSI [40].

Abbreviations: ITZ, itraconazole; TBZ, tebuconazole; n.d., not determined due to difficulties in identification or difficulties in sporulation.

### Minimum inhibitory concentrations in the fungal isolates from the plates containing 4 or 16 μg/mL of the triazoles

Table 2 shows the minimum inhibitory concentrations (MICs) of ITZ and TBZ in the isolates from the plates containing 4 or 16 μg/mL of ITZ or TBZ confirmed by the CLSI M38-A2 broth dilution method. *A*. *niger* (13L06I1) and *A*. *tubingensis* (27L05I1) each had an ITZ MIC of 2 μg/mL or greater (Table 2). In terms of their concentration in the air, these two isolates represent 0.4 CFU/m^3^ when averaged over all the sampling months and locations. For these two isolated strains, two types of ITZ-containing plates (4 and 16 μg/mL) were used requiring twice the sampling duration compared to the sampling duration used to quantify the concentration of total thermotolerant fungi using only one type of non-triazole-containing plate. A total of 25 thermotolerant fungal colonies were detected using only one type of non-triazole-containing plate (Table 1). Taking into consideration that twice the sampling duration was required to isolate these two strains, these potentially ITZ-resistant or intermediate strains represent 4% of the total thermotolerant fungi. All seven *P*. *variotii* strains analyzed had a TBZ MIC of 32 μg/mL or greater (Table 2).

## Discussion

This study quantified the concentrations of outdoor airborne thermotolerant fungi, which can be opportunistic infectious pathogens [30, 43, 44]. It was estimated that 0.17% of all airborne culturable fungi were thermotolerant. Remarkably, the number of cultured thermotolerant fungal colonies was higher on plates containing a small amount of ITZ (0.0625 and 0.25 μg/mL) than on non-triazole-containing plates (Fig 2). This could be stimulated fungal growth because of the low-dose exposure to triazoles. A similar tendency was reported for the plant pathogenic *Fusarium graminearum*, for which the biosynthesis of mycotoxin deoxynivalenol was induced in response to a sub-lethal exposure to prothioconazole [45]. It is possible that short-term variations in the ambient fungal concentrations influenced our observations because twelve consecutive air samplings (each lasting 2 or 5 min) were done. However, this is unlikely for the following reasons: i) the results are based on the cumulative values for 21 seasonal samplings; ii) large numbers of colonies (>50 CFUs) were observed only on the 0.0625 μg/mL ITZ plates but not on the non-triazole-containing plates (Table 1), and iii) both of the lowest ITZ concentrations (0.0625 and 0.25 μg/mL) showed a similar trend. Future studies are warranted to examine the causes of this tendency.

The fraction of thermotolerant fungal colonies observed on plates containing higher amounts of ITZ and TBZ (1, 4, and 16 μg/mL) was smaller than the fraction cultured on the non-triazole-containing plates (Fig 2), suggesting that growth was inhibited by the further addition of the triazoles. Though the mechanism of action varies by type of triazole and fungus [46], the chief mechanism is the inhibition of 14α-demethylation in the ergosterol synthetic pathway [20, 47]. The inhibition of ergosterol biosynthesis results in alteration of permeability and fluidity of fungal membrane [20]. A reduction of obtusifolione to obtusifoliol in sterol biosynthesis is another inhibitory mechanism of ITZ [46, 48]. No additional mechanism has been reported for TBZ [47]. The multiple modes of action for ITZ are thought to contribute to a broad spectrum of antifungal efficacies [49]. Overall, our results suggest that the growth of airborne fungi was mostly inhibited by the addition of more than 1 μg/mL of ITZ and TBZ in the plates (Table 1 and Fig 2).

Species were identified for fungi isolated from plates containing 4 and 16 μg/mL of ITZ and TBZ. The identified species included *A*. *fumigatus*, *A*. *niger*, *A*. *tubingensis*, and *P*. *variotii*. The reported spore diameters for *A*. *fumigatus* and *A*. *niger* are 2–3.5 μm and 3.5–5 μm, respectively [13]. Though data are unavailable for *A*. *tubingensis*, its spore size is expected to be similar to that of *A*. *niger* because *A*. *tubingensis*, a member of the *A*. *niger* species complex, is known to be morphologically indistinguishable from *A*. *niger* [15]. The spore size (W×L) of *P*. *variotii* is 3.5×5 μm [50]. Particles in these size ranges are inhalable and partly respirable by humans [14]. The inhalation of these pathogenic fungal spores is thought to cause pulmonary fungal infections [16]. Indeed, all of the *Aspergillus* species identified in this study are known to cause invasive pulmonary aspergillosis (IPA) [15, 16]. Cases of pulmonary infection by *Paecilomyces* species, including *P*. *variotii*, have also been reported [51, 52].

The MICs of ITZ and TBZ for the *Aspergillus* and *Paecilomyces* isolates were determined with the standard CLSI broth dilution method. Two potentially ITZ resistant or intermediate strains of *A*. *niger* (13L06I1) (MIC = 2 μg/mL) and *A*. *tubingensis* (27L05I1) (MIC = 4 μg/mL) were identified (Table 2). These two species, belonging to *Aspergillus* section *Nigri*, are known to have three different antifungal susceptibility patterns [53]. Though there is no clinical breakpoint established for fungi, these isolates are possibly ITZ resistant or intermediate given that MICs below 1 μg/mL are common for most *Aspergillus* spp. [40] with a slightly higher epidemiologic cutoff value (2 μg/mL) reported for the section *Nigri* [54, 55]. This study estimated that 0.17% of the total airborne culturable fungi were thermotolerant, among which an estimated 4% showed ITZ MICs of 2 μg/mL or greater. The concentration of these two isolates in the air was 0.4 CFU/m^3^. Inhalation is an important route of exposure because people inhale more than 10 m^3^ of air each day [56]. Though the concentration was low, the result may indicate a risk of inhalation exposure to airborne antifungal-resistant fungi from outdoor environments.

TBZ is used as an agricultural fungicide; therefore, its efficacy has been assessed mostly for fungal plant pathogens. The efficacy of agricultural fungicides is assessed in terms of the effective concentration which results in a 50% reduction of mycelial growth (EC_50_). One study reported an EC_50_ value of 8.09 μg/mL for a TBZ-resistant *Fusarium graminearum* strain [57]. Though a direct comparison is difficult between the MIC and EC_50_, the seven *P*. *variotii* isolates in this study appeared to be highly resistant to TBZ, i.e., MICs >32 μg/mL (Table 2). Taking into consideration that TBZ is not used as an antifungal medication and that *P*. *variotii* is not a known plant pathogen [58], the finding of *P*. *variotii* high resistance to TBZ may not be of importance in terms of the public health or agriculture. However, a recent study has shown the cross-resistance of medical and agricultural triazoles in clinical and environmental *A*. *fumigatus* isolates [26]. Though cross-resistance to ITZ was not found in the *P*. *variotii* isolates that exhibited a high resistance to TBZ in this study, further studies are warranted to monitor the emergence of cross-resistance in human fungal pathogens.

*Aspergillus fumigatus* is a primary and opportunistic fungal pathogen with known azole resistance mechanisms mediated by Cyp51, the efflux pump, and stress adaption [35, 36, 59]. Among the Cyp51-mediate mechanisms, a mutation in the number of 34-bp tandem repeats in the promoter region combined with a substitution at codon 98 in the *cyp51A* gene (TR_34_/L98H) was thought to be predominant in *A*. *fumigatus* [24, 25, 37]. This study did not detect the TR_34_/L98H mutants of *A*. *fumigatus* from the isolates collected from the outdoor air in the study area. This could be partly due to the limited number of *A*. *fumigatus* isolates (*n* = 3) analyzed in this study, with all of them having ITZ MICs smaller than 2 μg/mL. The TR_34_/L98H mechanism was initially reported in Europe [60], but it is expanding globally with reports from China, Iran, India, Kuwait, and Tanzania [22, 61, 62]. Because of the nature of the TR_34_/L98H mechanism with its environmental origin in *A*. *fumigatu*s [61], it is possible that TR_34_/L98H strains will emerge locally from the environment.

Triazoles are the most successful antifungal class both clinically and agriculturally; however, there are concerns over the emergence of resistant strains. Because of the use of triazoles throughout the world, it is important to monitor the emergence of triazole resistance in the environment. This study examined triazole susceptibility in pathogenic thermotolerant fungi isolated from the ambient air around the capital area of Seoul, South Korea. This study estimated that 0.17% of the total culturable fungi belong to the pathogenic thermotolerant taxa, among which the isolates of *A*. *niger* and *A*. *tubingensis* had ITZ MICs of 2 μg/mL or greater, and seven *P*. *variotii* isolates had TBZ MICs of 32 μg/mL or greater. The concentration of the two *Aspergillus* isolates with high ITZ MICs in the air was 0.4 CFU/m^3^. Though their abundance was low, our findings suggest the presence of airborne fungal strains with high MICs for medical and agricultural triazoles in outdoor environments. Inhalation is an important route of exposure because people inhale more than 10 m^3^ of air each day. Vigilance is preferred over monitoring for the emergence of triazole-resistant fungal pathogens in ambient outdoor air.
